# Elephant Driven Changes in Riverine Tree Density Exacerbated by Biological Infestation in Samburu and Buffalo Springs National Reserves, Kenya

**DOI:** 10.1002/ece3.72692

**Published:** 2025-12-12

**Authors:** Vincent Kipkazi, Eunice Kairu, Giacomo D'Ammando, George Wittemyer, Iain Douglas‐Hamilton, Festus W. Ihwagi

**Affiliations:** ^1^ Department of Zoological Sciences Kenyatta University Nairobi Kenya; ^2^ Save the Elephants Nairobi Kenya; ^3^ Department of Fish, Wildlife, and Conservation Biology and Graduate Degree Program in Ecology Colorado State University Fort Collins Colorado USA; ^4^ Department of Zoology University of Oxford Oxford UK

**Keywords:** debarking, insect infestation, *Loxodonta africana*, plant response, recovery

## Abstract

African elephants ( *Loxodonta africana* ) can profoundly impact the ecosystems in which they live and, therefore, are considered ecosystem engineers. Elephants break, push over, uproot, and de‐bark woody plants, which can threaten the survival of some tree species. In this study, we investigated the changes in the composition, structure, and de‐barking condition of riverine woodlands in the semi‐arid Samburu and Buffalo Springs National Reserves of northern Kenya, over a 16‐year period. Further, we assessed recruitment potential, the plant's response to debarking, and the subsequent disturbances by termites and woodborers. We conducted surveys of the woody plant community in nine 100 × 100 m riverine plots in 2007 and in 2023. All woody plants taller than 3 m with stem circumference over 6 cm were surveyed. Elephant debarking was assessed using a 0–5 scale: 0 (no debarking) to 5 (intensively debarked), based on the proportion of stem circumference debarked. We found that tree density declined between 2007 and 2023, with sapling density showing a marked reduction, indicating lower recruitment in 2023. Counts of trees across circumference classes differed between 2007 and 2023, with *Vachellia elatior* exhibiting a lower number of debarked individuals in 2023. Post‐debarking tree recovery was significantly influenced by debarking severity, insect infestation, and stem decay. Termite presence on debarked trees reduced recovery likelihood, while woodborers had no measurable effect. Additionally, stem decay was associated with reduced tree recovery potential. These findings highlight the compounding impact of biological infestation with elephant debarking on tree survival. Understanding the interconnected effects of elephants and other organisms on plant communities is important to inform future habitat management and possible interventions for ecosystem restoration.

## Graphical abstract

This study investigates the long‐term effects of elephant debarking on riverine woodland vegetation in northern Kenya, comparing data from 2007 and 2023. Findings reveal a decline in woody plant density and reduced tree recovery, especially in the presence of termites and stem decay. These results highlight the compounded ecological impact of elephants and other organisms on plant community structure and ecosystem health.

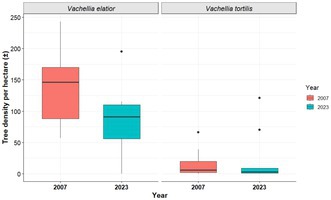

## 1 Introduction

**PLATE 1:**
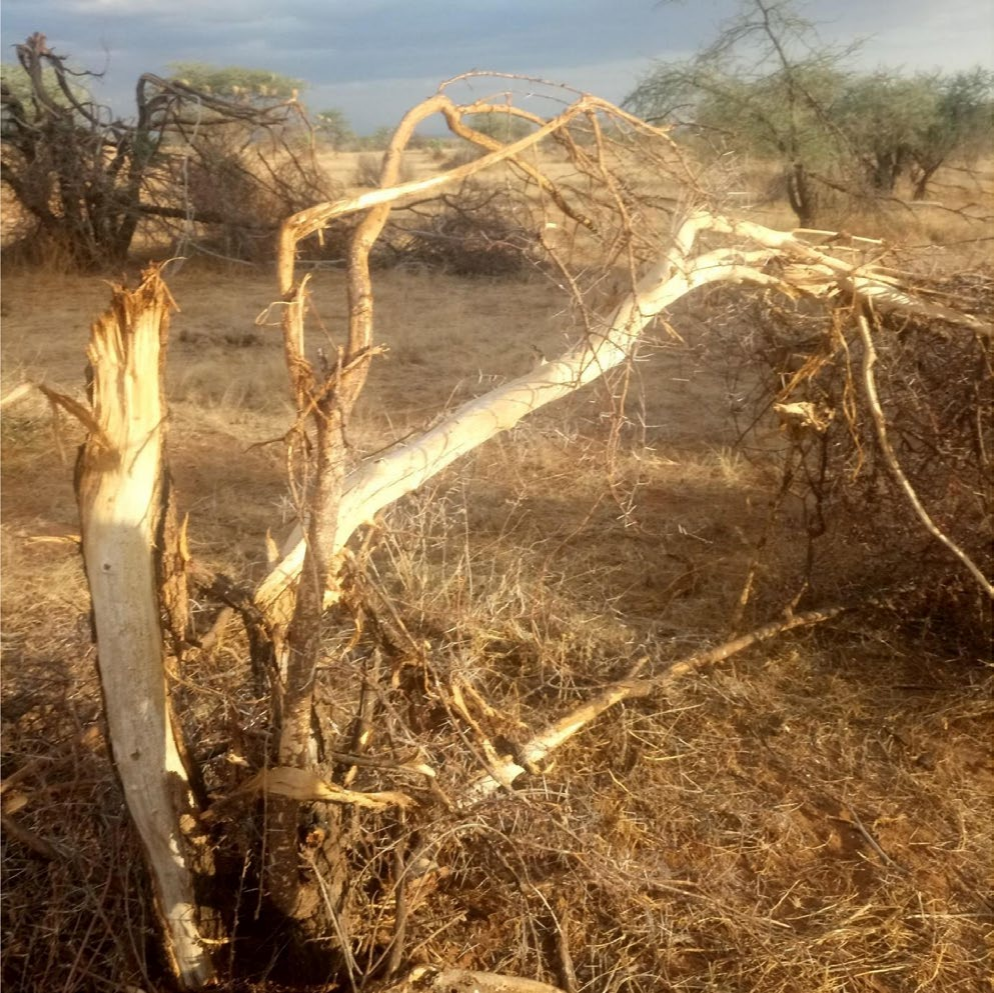
Severely debarked *Vachellia tortilis* in Samburu National Reserve.

African elephants ( *Loxodonta africana* ), the largest terrestrial herbivores (Owen‐Smith 1988), play a key role in shaping savanna ecosystems and are often considered as “ecosystem engineers” (Chomba and Banda 2016). This is largely due to their destructive feeding habits, which have a profound influence on the distribution, richness, and diversity of the multiple plant species making up their diet (Nasseri et al. 2010; Pringle et al. 2015). In some cases, elephant foraging behavior can also alter vegetation structure to the point of facilitating the conversion of woodland into grassland, with cascading effects on community‐ and ecosystem‐level processes, as well as on the effectiveness of protected areas at preserving existing biodiversity (Croze 1974; Chomba and Banda 2016; Glover 1963; Osborn 2002; Pamo and Tchamba 2001). A particular cause of concern to protected area managers is the de‐barking of trees by elephants (Plate 1), which in severe cases can lead to mass mortalities of trees and even threaten the persistence of some tree species (Chomba and Banda 2016; Ihwagi et al. 2010). This form of damage often occurs alongside the intensive use of individual trees, which can suppress regeneration, reduce sapling survival, lower tree density, and cause long‐term alterations to tree structure (Barnes 1985; Guldemond 2006), particularly in areas with high elephant numbers (Kohi et al. 2011; Wahungu et al. 2011). Elephant impacts can also diminish habitat quality for other species—for example, by influencing predation risk and resource availability—ultimately contributing to broader ecosystem degradation (Valeix et al. 2011).

As mixed feeders with a hindgut fermentation system, elephants are capable of utilizing a wide range of forage types, including grasses, leaves, twigs, roots, fruits, flowers, and bark (Laws 1970; Ihwagi et al. 2010; O'Connor and Page 2014; Seloana et al. 2018). Selection for different forage types varies seasonally due to seasonal fluctuations in plant nutrient content, biomass, and digestibility typical of African savannahs (Ihwagi et al. 2012; Pamo and Tchamba 2001). During the wet season, elephants predominantly graze on young, nutrient‐rich grasses. However, as the dry season advances and the nutritional quality of grasses declines and green forage becomes depleted (Barnes 1982; Holdo 2003; Valeix et al. 2011), they progressively shift to browsing on woody vegetation (Nasseri et al. 2010; Valeix et al. 2011; Gill et al. 2023). Tree bark is an important dry‐season resource because it contains essential nutrients and sugars, while generally having lower concentrations of secondary metabolites (Holdo 2003; Seloana et al. 2018). Consequently, bark consumption—and thus debarking—typically intensifies during periods when bark nutritional value peaks, particularly when sap flow facilitates leaf emergence and flowering (Barnes 1982).

**PLATE 2:**
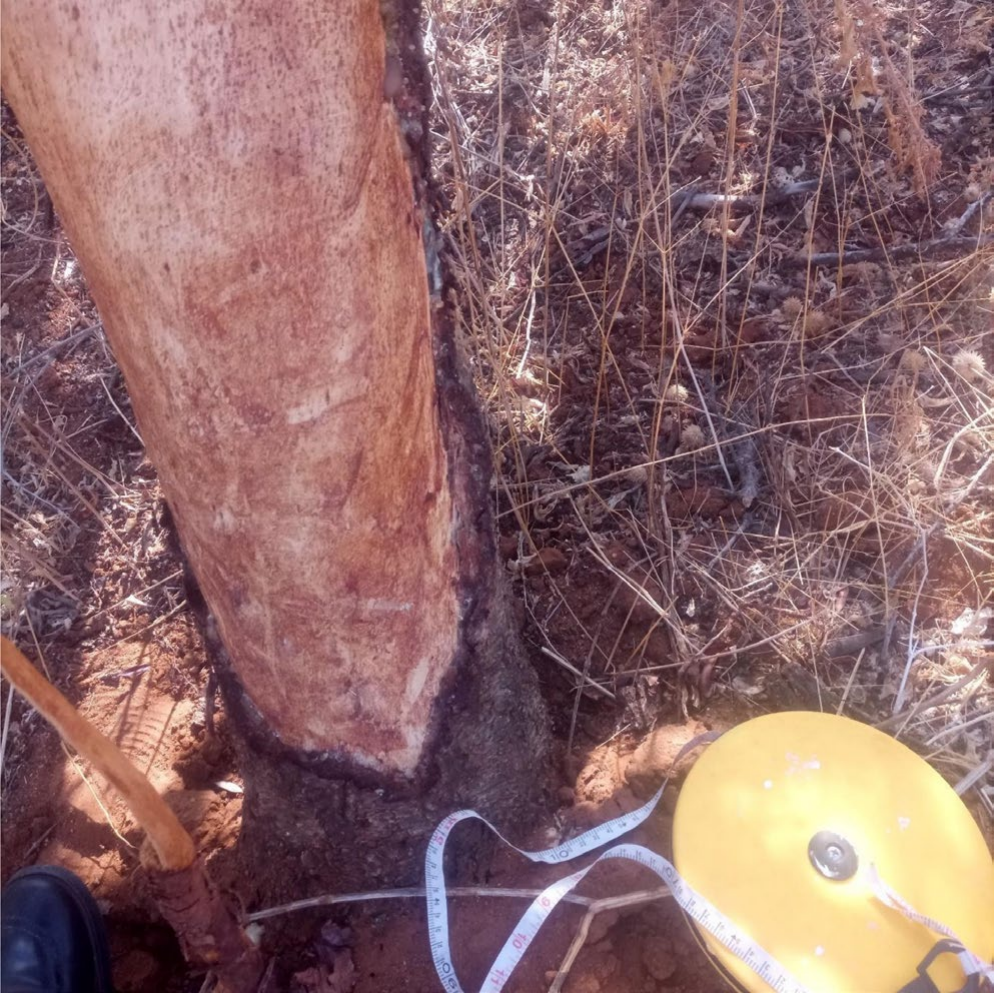
Severely debarked *Vachellia elatior* in Samburu National Reserve‐ layers indicating subsequent debarking on an earlier regrown bark.

Despite the key nutritional value of bark for elephants during resource‐scarce periods, intensive and repeated debarking can severely compromise tree health and structure (Pamo and Tchamba 2001; Plate 2). Without sufficient recovery time, trees may dry out, decay, and eventually die (Delvaux et al. 2009). Exposed tissues increase vulnerability to fire, pathogens, and insect infestations, particularly by termites and woodborers that exploit debarked areas for food and nesting (Helm et al. 2011; Shannon et al. 2011; Joseph et al. 2011; N'Dri et al. 2011). Termite infestations can hollow out stems, further weakening trees and amplifying susceptibility to mechanical damage from elephants, other animals, or wind (Werner et al. 2008). Debarking also facilitates entry of fungal and bacterial pathogens, reducing a tree's ability to recover (Gauthier et al. 2015). However, there is relatively little information on how debarking and parasites/pathogens interact to affect vegetation structure and composition over long periods of time. This is really very important to help us understand the knock‐off effect it can have on other species and how such risks can be curbed to help trees recover while simultaneously securing elephant survival.

In this study, we aimed at quantifying changes in woodland composition, structure, and debarking condition in a semi‐arid savanna ecosystem of northern Kenya by comparing current vegetation data with a 2007 baseline survey. We also assessed plant recovery following elephant debarking. We specifically focused on two species of trees (*Vachellia elatior* and *Vachellia tortilis* ) to monitor changes in comparison with the baseline because they were common along the riverine forest and were the main tree species preferred by elephants for debarking in 2007 (Ihwagi 2007). Our first hypothesis was that elephants had modified the structure and composition of the riverine forest compared to the baseline survey. Accordingly, we predicted that the density of trees and the diversity of tree species had reduced over the period between 2007 and 2023. We also predicted that the proportion of debarked trees would have been higher in 2023 compared to 2007. Second, we hypothesized that elephants had negatively affected the recovery of the riverine forest between 2007 and 2023. If elephants had indeed acted as the main inhibitors of recovery, we predicted that the recruitment of saplings for the two tree species favored by elephants would have been lower in 2023 compared to 2007. We also predicted that the recovery of trees post‐debarking was reduced in relation to the severity of debarking. Finally, we tested the hypothesis that recovery post‐debarking was inhibited by the presence of insect damage in addition to the elephant debarking.

## 2 Materials and Methods

### 2.1 Study Area

**FIGURE 1:**
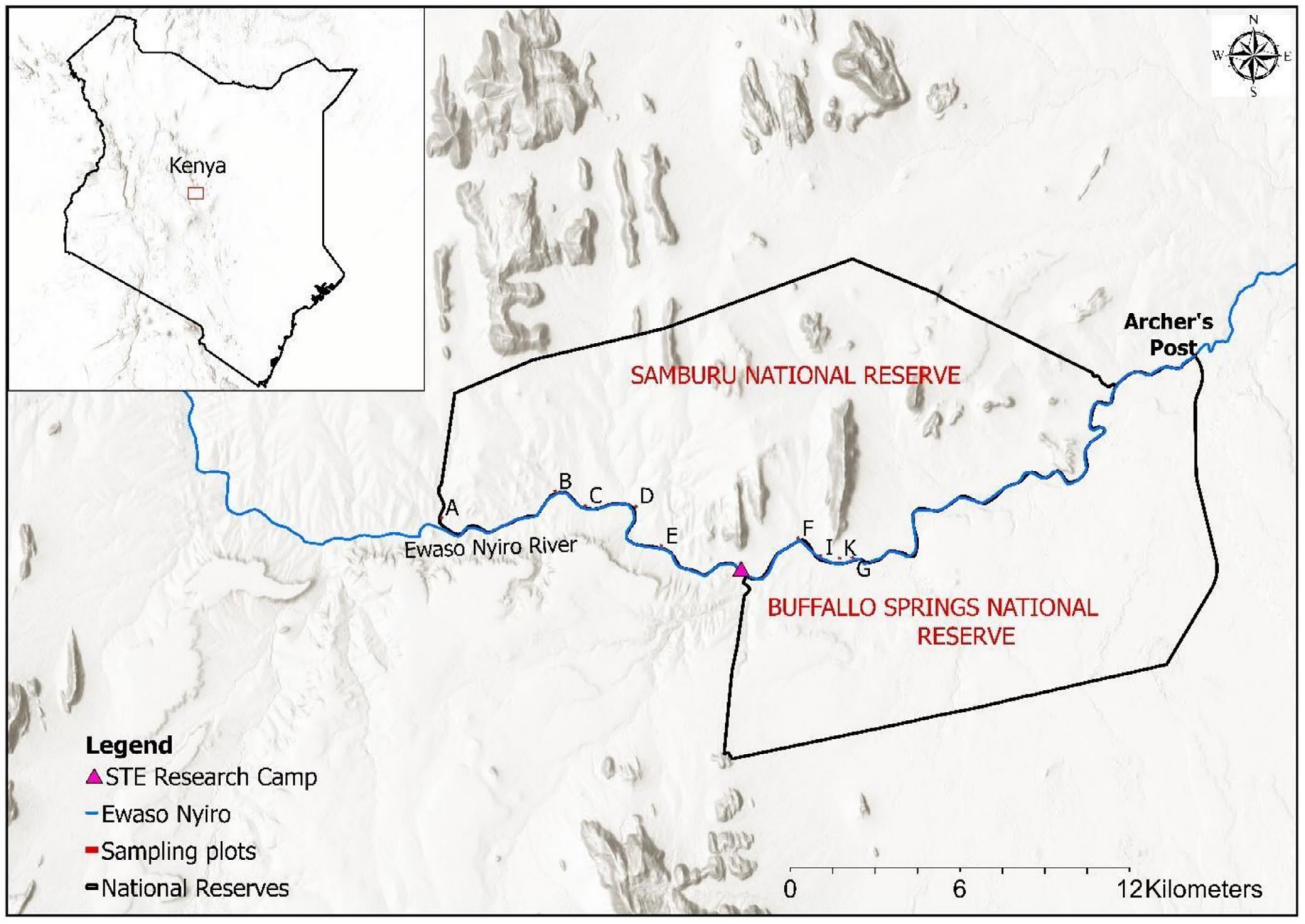
The location of Samburu and Buffalo Spring National Reserves in Northern Kenya.

This study was conducted in the Samburu and Buffalo Springs National Reserves of northern Kenya (Figure 1). The reserves have a combined area of approximately 336km^2^ (Wittemyer et al. 2005) and lie opposite each other at 0°30′ N, 37°30′ E. They are separated by the Ewaso Nyiro River, the only permanent water source in the landscape and a focal area for elephants (Ihwagi 2007). Rainfall in the region averages approximately 350 mm per year and occurs during biannual rainy seasons, generally falling in April/May and November/December (Ihwagi et al. 2012). The vegetation in the reserves comprises riverine forest along the Ewaso River dominated by *Vachellia elatior* (previously known as *Acacia elatior*) and doum palms ( *Hyphaene thebaica* ). *Vachellia elatior* is largely confined to areas adjacent to the river, whereas *Vachellia tortilis* occurs both within riverine zones and in more distant woodland areas. Since the focus of this study was on riverine vegetation, only trees occurring within the riverine forest were considered.

### 2.2 Data Collection

#### 2.2.1 Sampling Plots

Nine 100 m x 100 m riverine sampling plots were established in 2007 in the riverine forest along the Ewaso Nyiro river (Ihwagi et al. 2010). These plots were revisited and resampled in 2023 using the same method. The GPS waypoints of the 2007 plots were keyed into a handheld GPS device (Garmin 65 s), allowing navigation to the plot's corners. The corners of the plots were re‐marked using stones and dead logs painted white with environmentally friendly water‐based paint prior to surveying. Plots were visited between the 7th of August and the 8th of December 2023. Each plot was visited once every 2 weeks, for a total of nine visits per plot during the study period. Revisits were aimed at assessing recovery in trees damaged by elephants.

### 2.3 Assessment of Woody Plant Composition and Structure

In each plot, we first mapped each tree using a handheld GPS (Garmin 65 s). Each tree was identified to the species level. Trees were defined as individuals exceeding 3 m in height and having a circumference of at least 6 cm (Vesey‐FitzGerald 1973). The circumference of each tree was measured in centimeters at breast height (DBH) using a tape measure. The count of all species recorded was obtained to determine their abundance in each plot. For the two most common tree species in the plots, namely *Vachellia elatior* and *V. tortilis* , circumference measurements were grouped into 40‐cm size classes, and the number of trees in each class was quantified to evaluate changes in size structure. These species were also selected because they were the main targets of elephant debarking in a 2007 study (Ihwagi 2007). We mapped and assessed saplings of both species within the plots. Saplings were defined as individuals with stem circumferences between 6 and 46 cm. Trees in this size class were excluded from the tree‐density calculations but mainly used when calculating sapling density.

### 2.4 Assessment of Bark Utilization by Elephants

**PLATE 3:**
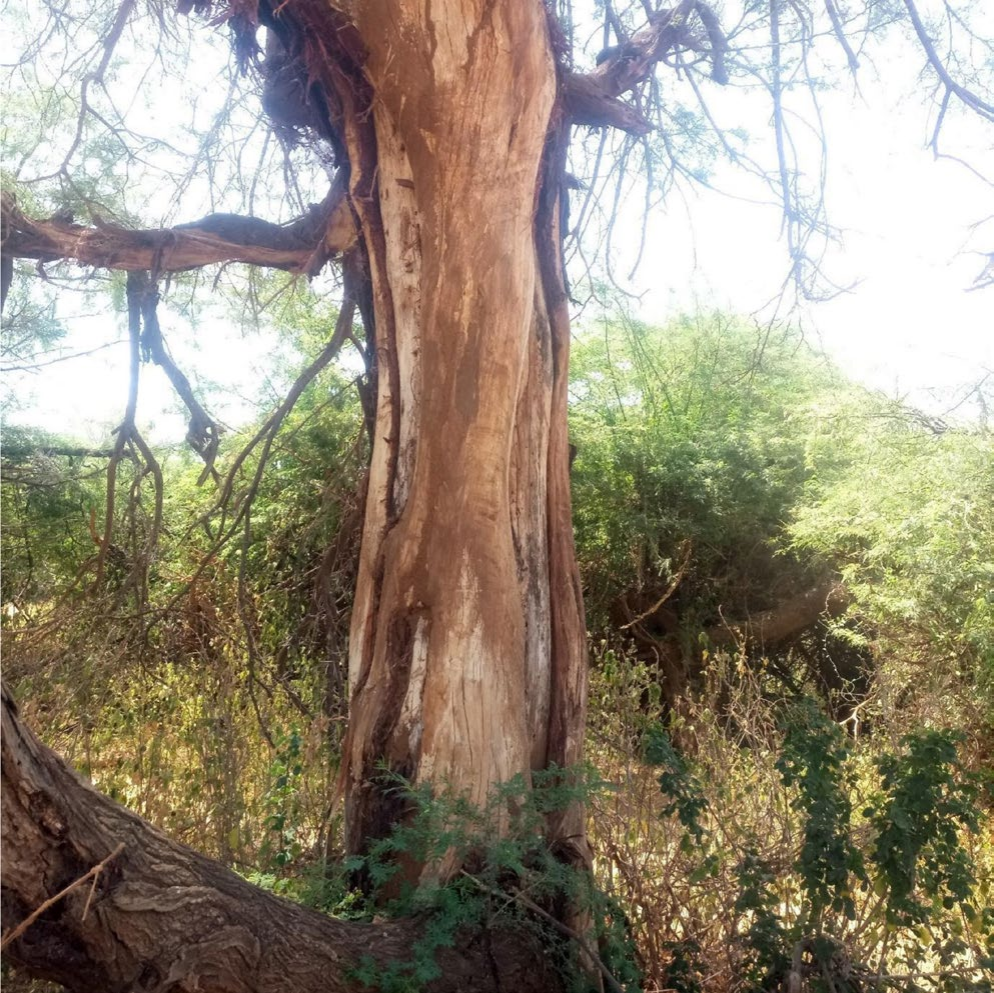
Freshly debarked *Vachellia elatior* tree in Samburu, exuding gum at the wound edges and showing elephant's tusk marks on the stem.

During each plot visit, we examined every tree for signs of elephant debarking, and we visually estimated the debarked proportion of each tree stem. Both standing trees and those that had been pushed over by elephants but remained alive were considered available for debarking. The visual estimate was based on a six‐point scale adapted from Walker (1976), and originally used in the 2007 survey (Ihwagi 2007), expressing the proportion of debarking as: 0 = 0%; 1 = 1%–25%; 2 = 26%–50%; 3 = 51%–75%; 4 = 76%–99%; and 5 = 100%. According to this scale, a fully utilized tree with complete ring debarking has a score of 5, and an unutilized tree has a score of zero (Table S1). Debarking was classified as old or recent. Signs of old elephant bark utilization were identified by loose fibers and dry, hardened sap at the wound site. In contrast, recent debarking was recognized by moist or sticky sap, bright wound surfaces, and firmly attached bark fibers. Elephant damage is distinctive from that of other herbivores (Jachmann and Bell 1985; Boundja and Midgley 2010) due to the visible elephant tusk marks on tree branches and stems (Plate 3).

### 2.5 Tree Response to Debarking

**PLATE 4:**
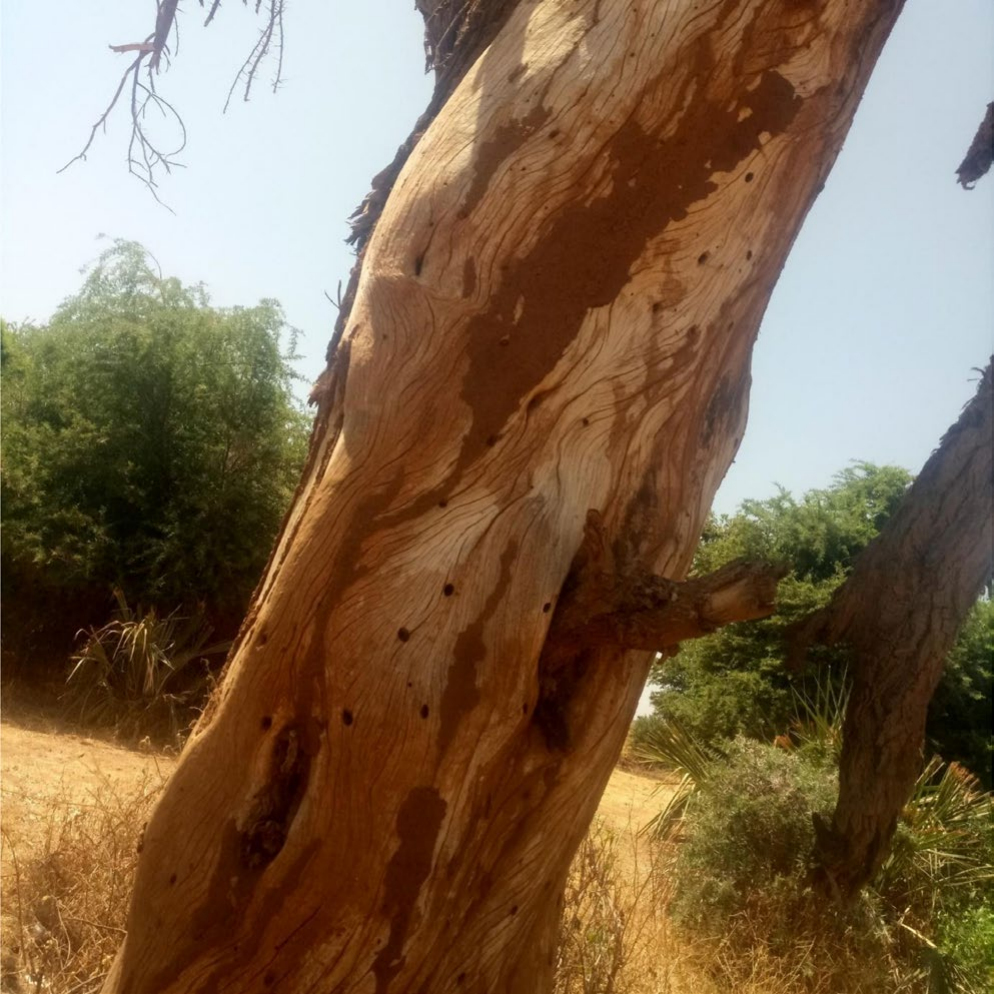
Termites and woodborers infestation along a debarked *Vachellia tortilis* stem in Samburu National Reserve.

We recorded any overall physical changes on debarked trees to assess whether debarked trees were recovering or not. The debarked sites on each tree were assessed for the presence of callus formation, coppicing, or decay of the stem. Callus formation refers to the regrowth of the bark over a wounded area of the stem (Thompson et al. 2022). Coppicing is the development of new shoots from the remaining stem, rootstock, broken branches, or the base of the stem (Gadd 2002; Tweheyo et al. 2013). Indicators of stem decay include open cavities, wood that is spongy and soft, and the presence of fungus or white grubs at wound sites—as specified by Gauthier et al. (2015). Most of the trees assessed for recovery response only showed signs of old damage (93%), while only a small fraction (7%) had been recently damaged (Figure S2). The newly debarked trees therefore were omitted, and the age of damage was thus not included in our analyses due to the very small sample size of recently damaged trees. All trees sampled were also examined for the presence of biological infestation by termites and woodborers. Termite infestation was identified by the mud galleries that run upwards from the foot of the tree (Plate 4). The presence of woodborers was identified by holes in tree stems, boring dust, feeding galleries beneath the bark, or the presence of the woodborer itself.

### 2.6 Data Analyses

We first calculated overall tree density (number of trees per plot area) per hectare in 2023 and compared it with tree density in 2007. Tree density per hectare for the two common species was calculated as tree counts per plot area and compared between 2007 and 2023. Data were first assessed for normality and homoscedasticity using both graphical (Q‐Q plots) and numerical methods (Shapiro–Wilk test; Mishra et al. 2019). A Shapiro–Wilk *p*‐value < 0.05 indicated non‐normality. Homogeneity of variance was evaluated using the *F*‐test and Levene's test, with *p* < 0.05 indicating unequal variances. Statistical test selection was based on whether these assumptions were met. A two‐sample *t*‐test was used to compare overall tree densities between 2007 and 2023. Species‐specific comparisons were conducted using two‐sample *t*‐tests for *Vachellia elatior* and Wilcoxon rank‐sum tests for *Vachellia tortilis* . For all other species, differences in densities between 2007 and 2023 were calculated but not compared statistically (due to the small sample sizes). Sapling densities were also compared between 2007 and 2023 to evaluate recruitment potential, with overall and species‐specific (for *V. elatior* and *V. tortilis* ) sapling densities again analyzed using two‐sample *t*‐tests.

To assess whether the number of trees in each debarking category differed between 2007 and 2023, we analyzed tree count as the response variable using a two‐way analysis of variance (ANOVA). The explanatory variables were year (two levels: 2007 and 2023) and debarking category (six levels: 0%—No damage, 1%–25%, 26%–50%, 51%–75%, 76%–99%, 100%—Ringed debarked), including their interaction (year × category). Analyses were conducted separately for each species (*Vachellia elatior* and *V. tortilis* ). The same approach was applied to evaluate whether the number of trees debarked (response variable) differed across circumference size classes and years, with circumference size class and year as explanatory variables, including their interaction. Where violations of normality or homogeneity of variance were detected, tree counts were transformed using ln (*x* + 1) prior to analysis to meet ANOVA assumptions.

Generalized linear mixed effects models (GLMM: binomial distribution) were used to model the recovery ability of the two common and most preferred woody plants (*Vachellia elatior* and *Vachellia tortilis* ) as a function of the debarking categories, circumference size, biological infestation, species of pest (termites or wood borers), and whether the tree stem was decaying or not. The recovery response was coded as a binary variable, with 1 indicating recovered trees and 0 indicating non‐recovered trees, enabling the use of a binomial distribution in the model. Among the covariates, both the debarking category (0%—No damage, 1%–25%, 26%–50%, 51%–75%, 76%–99%, 100%—Ringed debarked) and pest species (none, both, woodborers, termites) were treated as categorical variables. Decay and infestation were each coded as binary variables, with a value of 1 indicating presence (decay or infestation) and 0 indicating absence. In all the models, plot ID was modeled as a random effect. Model selection was done using multi model inference under MuMIn Package in R. Model assumptions were assessed using the DHARMa package in R (Hartig 2022), which simulates scaled residuals to diagnose fit in mixed‐effects models. Residual diagnostics were performed using the simulate Residuals() function applied to the fitted model, and plots were visually inspected for deviations from homoscedasticity, independence, and distributional assumptions. No significant violations were detected (Figure S4). Models were ranked using Akaike's Information Criterion—AIC (Burnham and Anderson 2002), and model averaging was considered when competing models had AIC < 2 (Arnold 2010; Table S2). All analyses were conducted in R using the ‘lme4’ package with the function glmer() (R Core Team 2021).

## 3 Results

### 3.1 Changes in the Composition and Structure of Riverine Woodland

**FIGURE 2:**
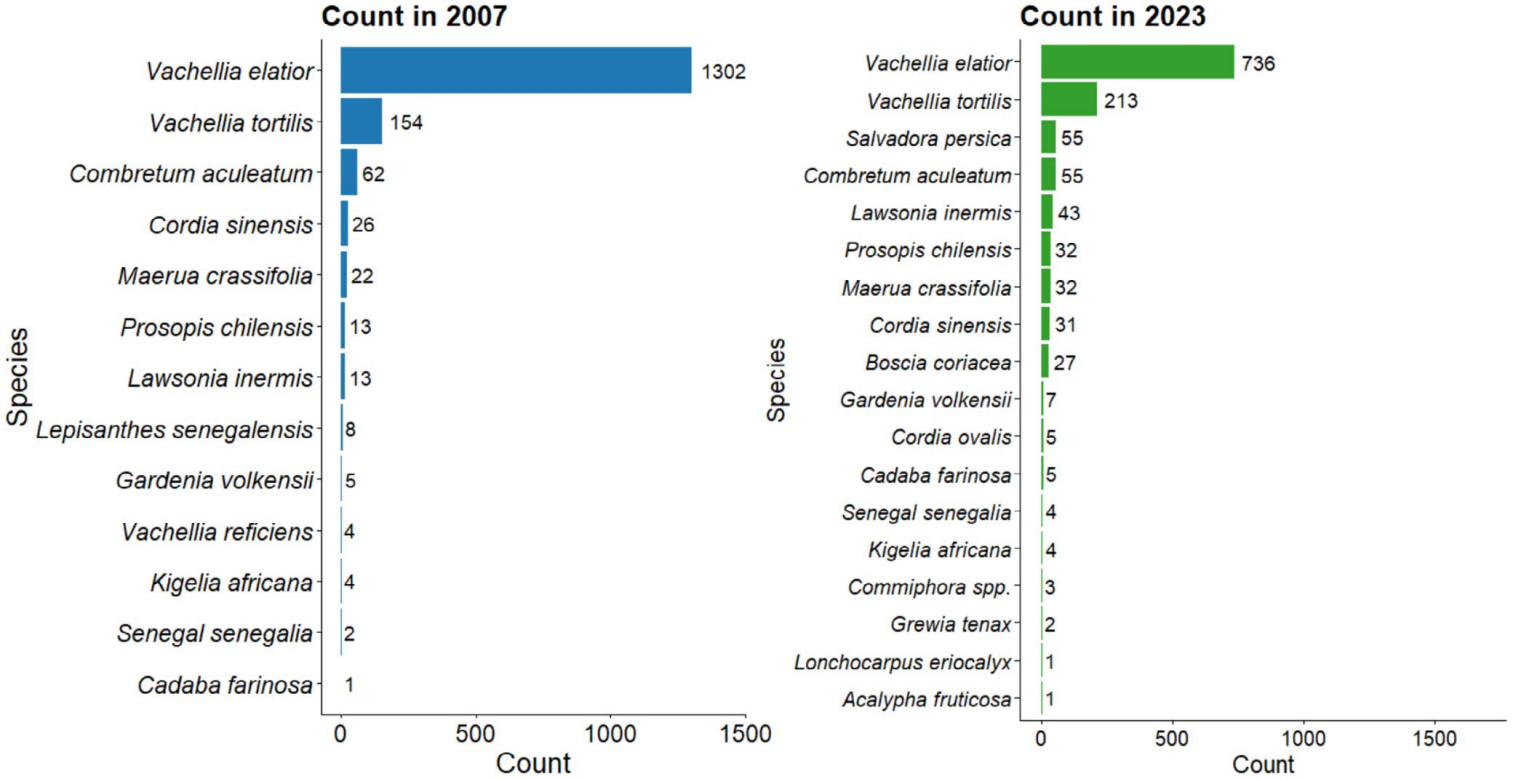
Abundance of tree species recorded in riverine plots in 2007 and 2023.

**FIGURE 3:**
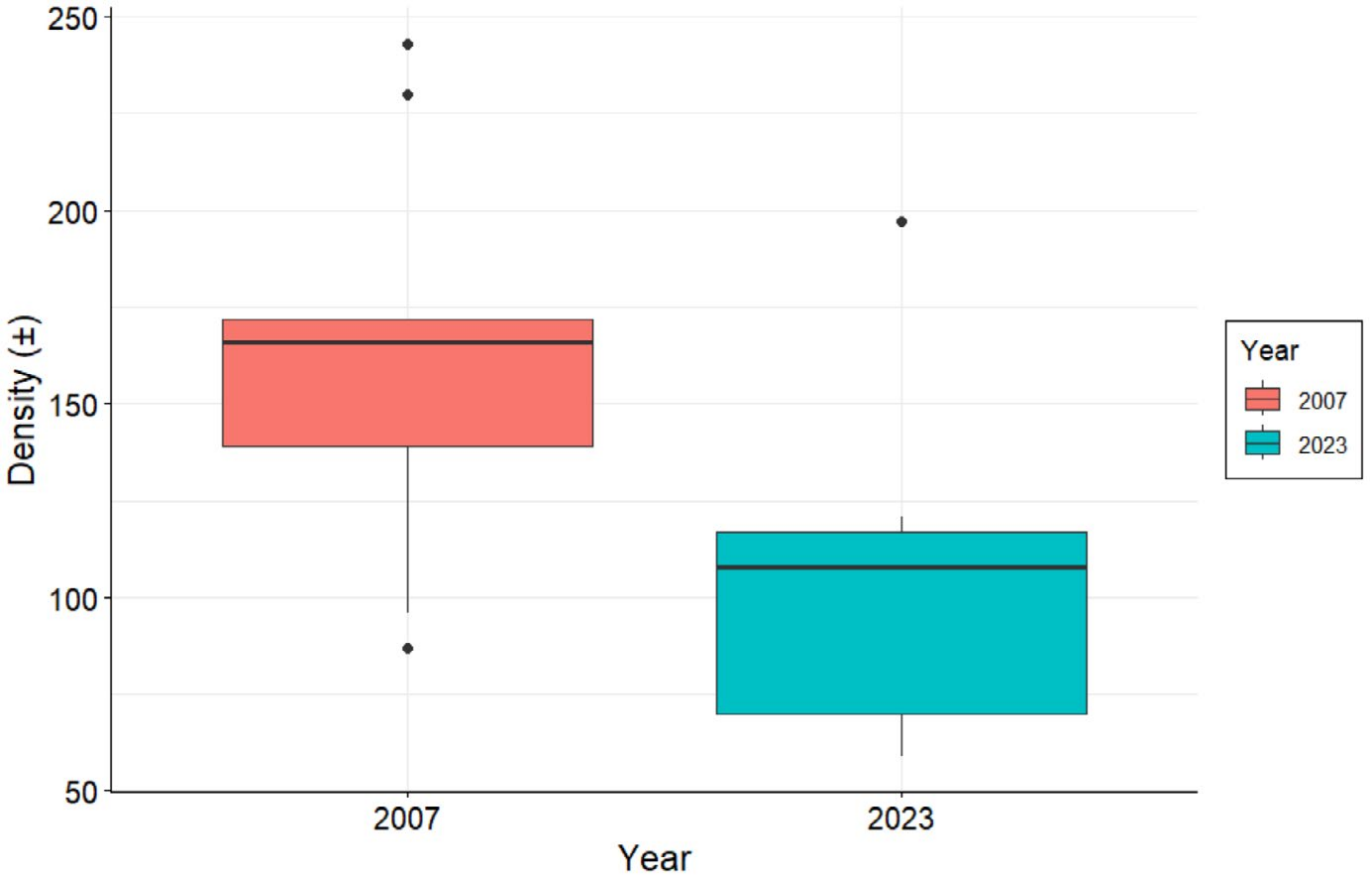
Tree mean densities in 2007 (*n* = 1456) and 2023 (*n* = 949).

**FIGURE 4:**
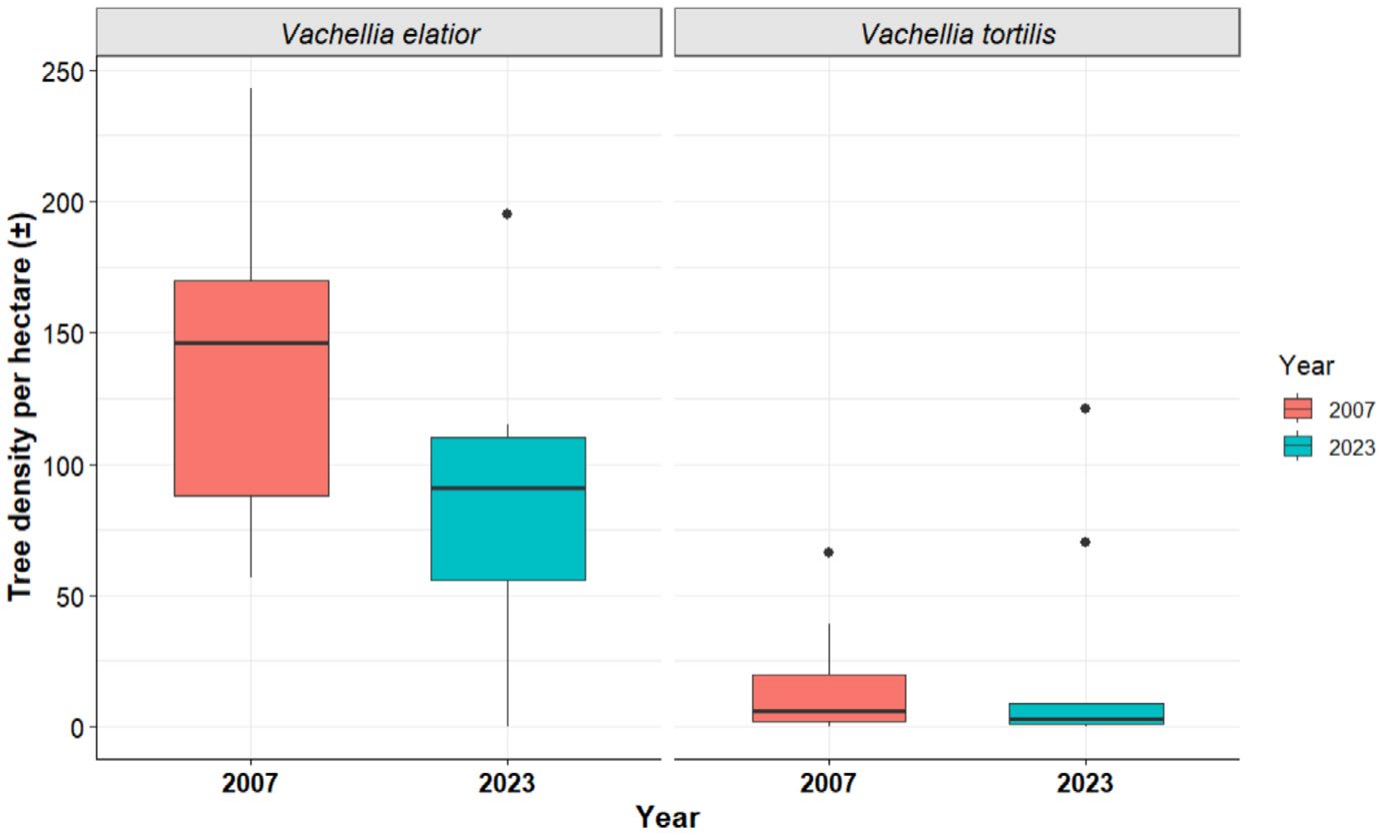
Density of *Vachellia elatior* and *Vachellia tortilis* trees recorded in 2007 and 2023. Sample sizes for *V. elatior* were *n* = 1302 (2007) and *n* = 736 (2023), and for *V. tortilis* were *n* = 154 (2007) and *n* = 213 (2023).

Tree species richness increased from 13 species in 2007 to 18 species in 2023 (Figure 2). Among the species recorded in 2023, the abundance of *Prosopis chilensis* , *Salvadora persica*, *Cordia sinensis* , *Boscia coriacea*, and *Lawsonia inermis* was higher than in 2007 (Figure 2). *Vachellia elatior* constituted 81% of sampled trees in 2007 and 59% in 2023, while *Vachellia tortilis* represented 10% in 2007 and 17% in 2023 (Figure 2). Overall tree density was significantly lower in 2023 than in 2007 (2007: 162 ± 17 SE trees ha^−1^; 2023: 105 ± 13 SE trees ha^−1^; *t* (16) = 2.53, *p* = 0.022; Figure 3). The mean density of *V. elatior* was lower in 2023 (81 ± 20 SE trees ha^−1^; *t* (16) = 2.16, *p* = 0.046; Figure 4) than in 2007 (145 ± 21 SE trees ha^−1^)—however, significance was close to the 0.05 threshold. *Vachellia tortilis* , in contrast, showed no significant difference in density between 2007 (17 ± 7 SE trees ha^−1^) and 2023 (23 ± 14 SE trees ha^−1^; *W* = 43.5, *p* = 0.824; Figure 4).

**FIGURE 5:**
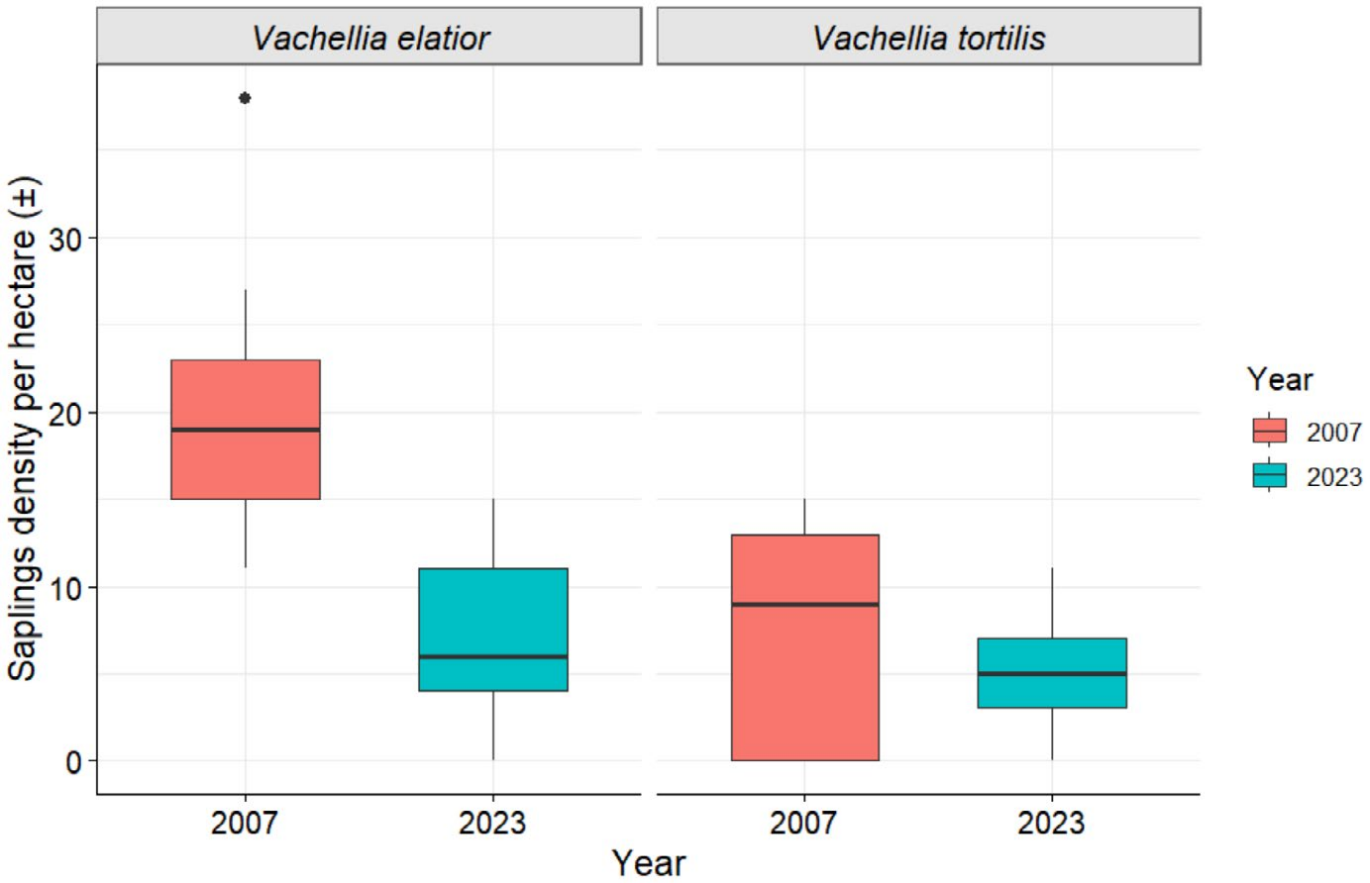
Density of *Vachellia elatior* and *Vachellia tortilis* saplings recorded in 2007 and 2023. Sample sizes were *n* = 187 (2007) and *n* = 61 (2023) for *V. elatior* , and *n* = 65 (2007) and *n* = 45 (2023) for *V. tortilis* .

The density of *V. elatior* saplings was significantly lower in 2023, with a mean density of 21 ± 3 SE saplings ha^−1^ in 2007 compared to 7 ± 2 SE saplings ha^−1^ in 2023 (*t* (16) = 4.30, *p* = 0.0005; Figure 5). In contrast, the density of *V. tortilis* saplings showed no significant difference between 2007 (7 ± 2 SE saplings ha^−1^) and 2023 (5 ± 1 SE saplings ha^−1^; *t* (16) = 0.90, *p* = 0.384; Figure 5).

**FIGURE 6:**
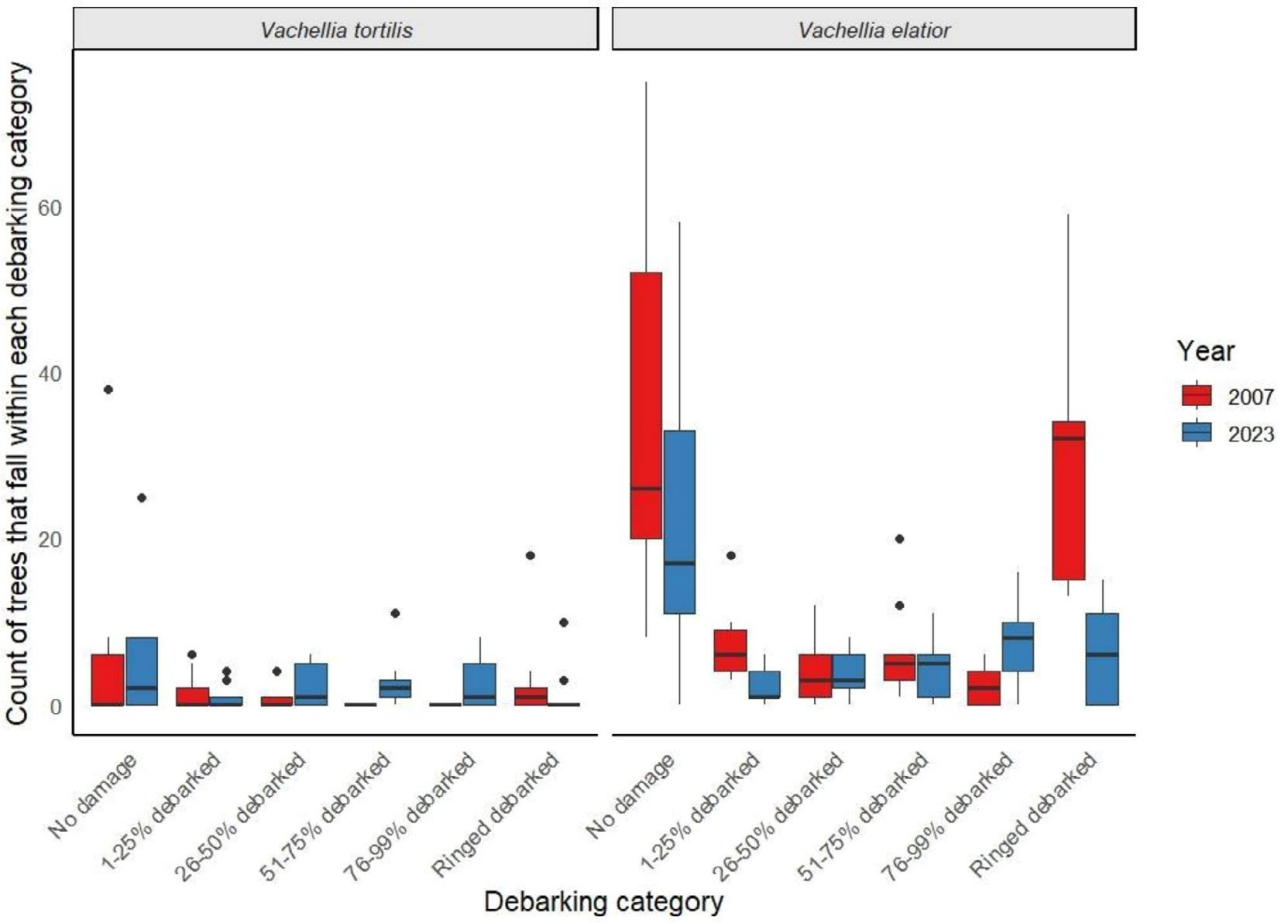
Count of *Vachellia tortilis and Vachellia elatior* in each debarking category in 2007 ( *V. elatior* *n* = 1302, *V. tortilis* *n* = 154) and 2023 ( *V. elatior* *n* = 736, *V. tortilis* *n* = 213).

**FIGURE 7:**
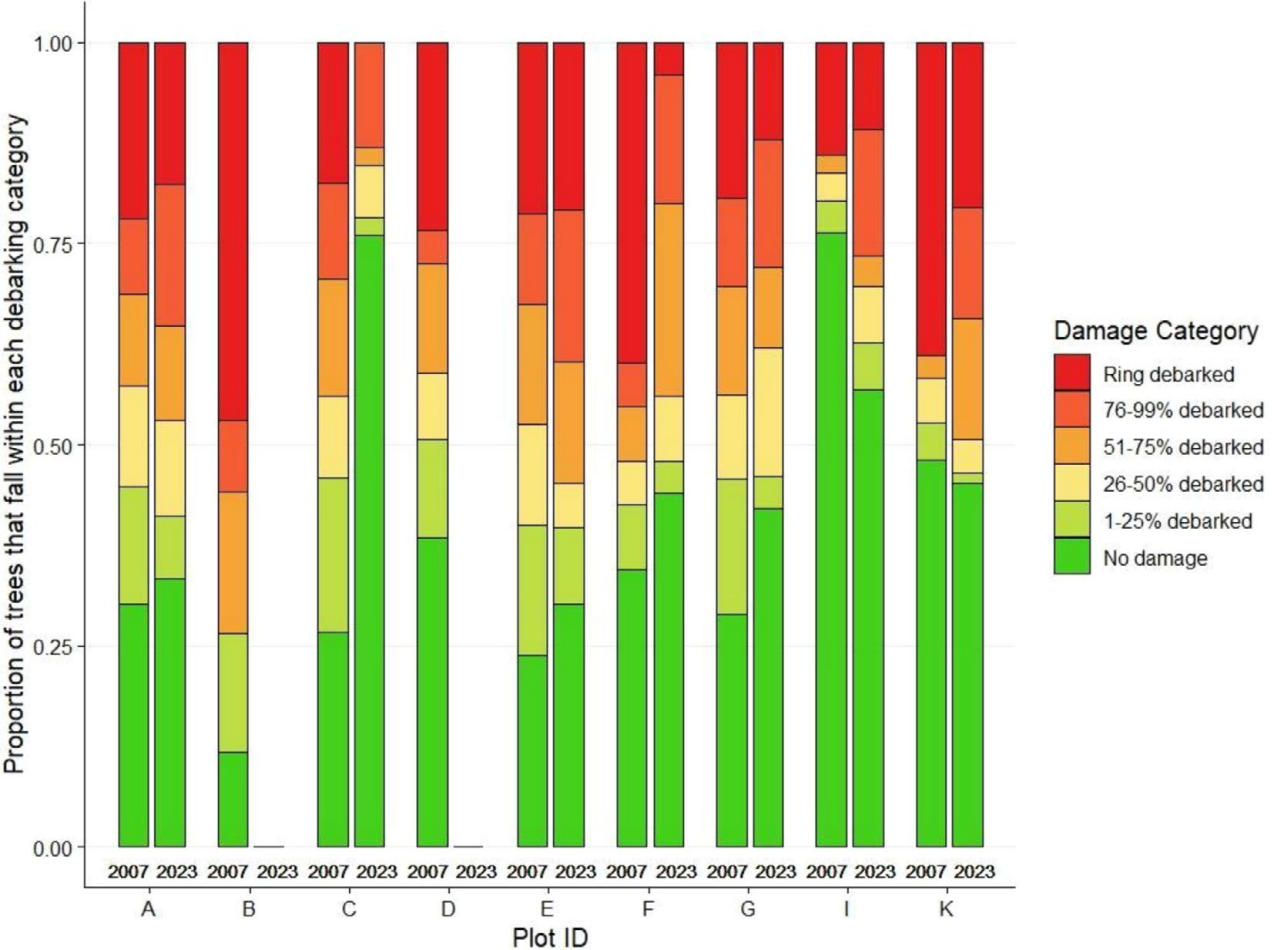
Proportion of *Vachellia elatior* that falls within each debarking category in 2007 (*n* = 1302) and 2023 (*n* = 736).

**FIGURE 8:**
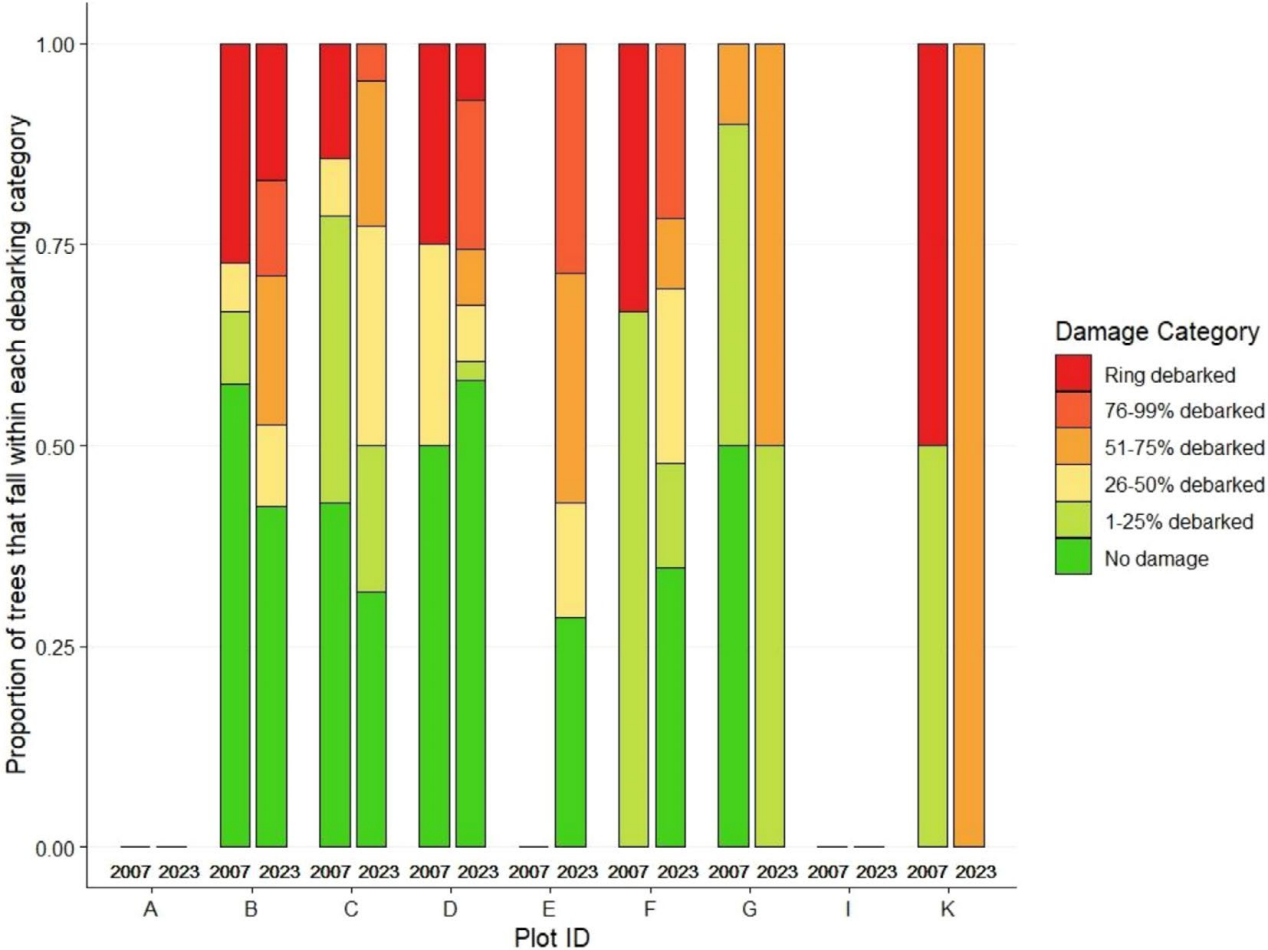
Proportion of *Vachellia tortilis* that falls within each debarking category in 2007 (*n* = 154) and 2023 (*n* = 213).

**FIGURE 9:**
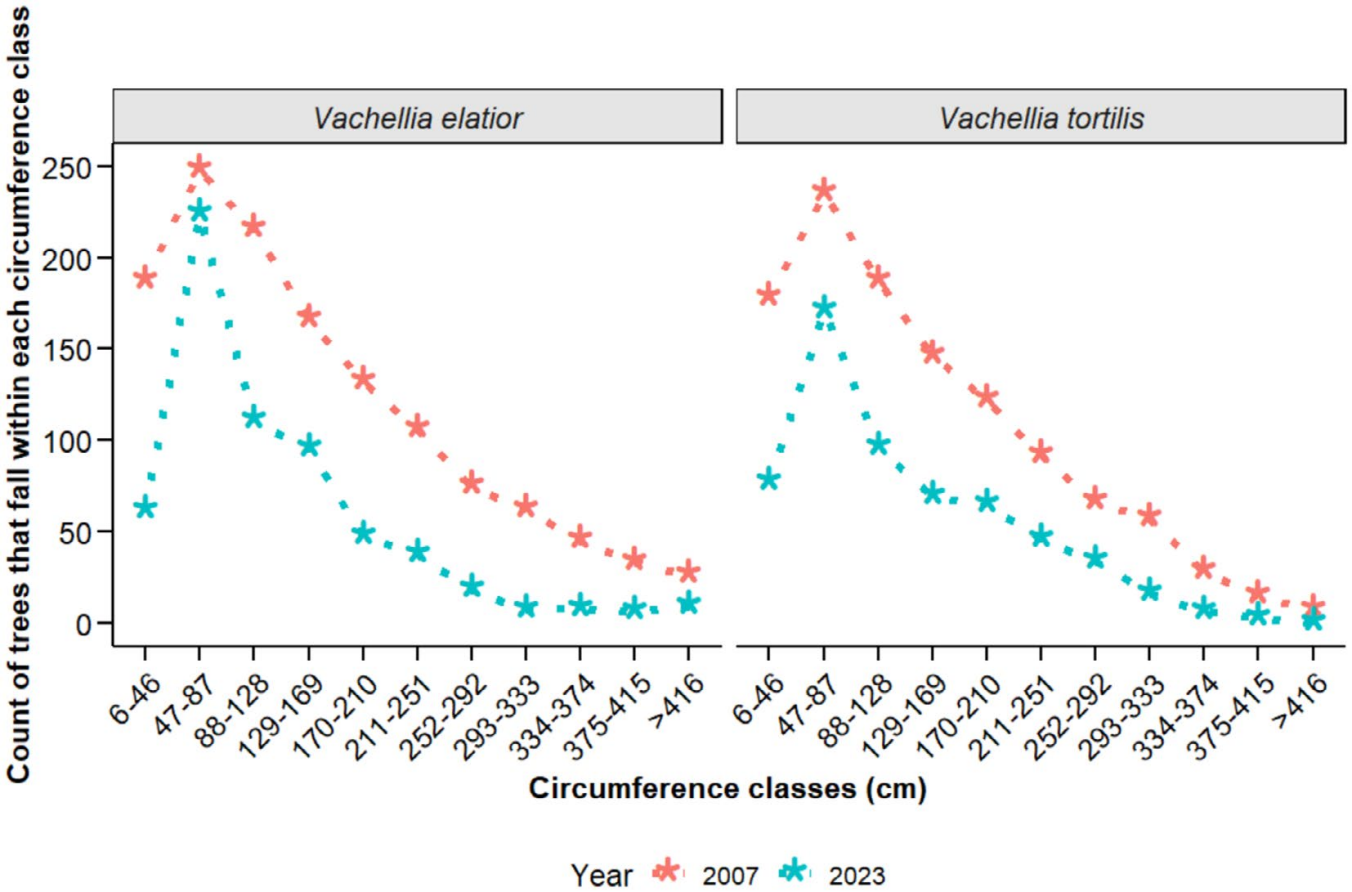
Number of trees recorded in each circumference class in 2007 and 2023. Sample sizes for *Vachellia elatior* were *n* = 1302 (2007) and *n* = 736 (2023), and for *Vachellia tortilis* were *n* = 154 (2007) and *n* = 213 (2023).

The number of *Vachellia elatior* trees debarked differed significantly between years and among damage categories (Year: *F*_1,96_ = 11.89, *p* = 0.0008; Category: *F*_5,96_ = 9.68, *p* < 0.05; Figures 6 and 7), with a significant year × category interaction (*F*_5,96_ = 4.53, *p* = 0.0009), showing that the number of trees debarked across categories was lower in 2023 than in 2007 (Figure 6). In contrast, *Vachellia tortilis* showed only a weak effect of year (*F*_1,96_ = 4.07, *p* = 0.046), with no significant differences among categories or year × category interaction (Category: *F*_5,96_ = 1.70, *p* = 0.14; Interaction: *F*_5,96_ = 1.65, *p* = 0.15) (Figures 6 and 8). Tree damage also varied across circumference classes for both species, with the highest numbers of damaged trees occurring in intermediate size classes (47–210 cm) ( *V. elatior* : Year *F*_1,110_ = 49.03, *p* < 0.05; Size class *F*_10,110_ = 10.78, *p* < 0.05; *V. tortilis* : Year *F*_1,110_ = 14.77, *p* < 0.05; Size class *F*_10,110_ = 17.29, *p* < 0.05; Figure 9). The year × size‐class interaction was not significant for either species ( *V. elatior* : *F*_10,110_ = 1.34, *p* = 0.218; *V. tortilis* : *F*_10,110_ = 0.54, *p* = 0.86).

### 3.2 Woody Tree Response to Debarking Resulting From Damage by African Elephants

**FIGURE 10:**
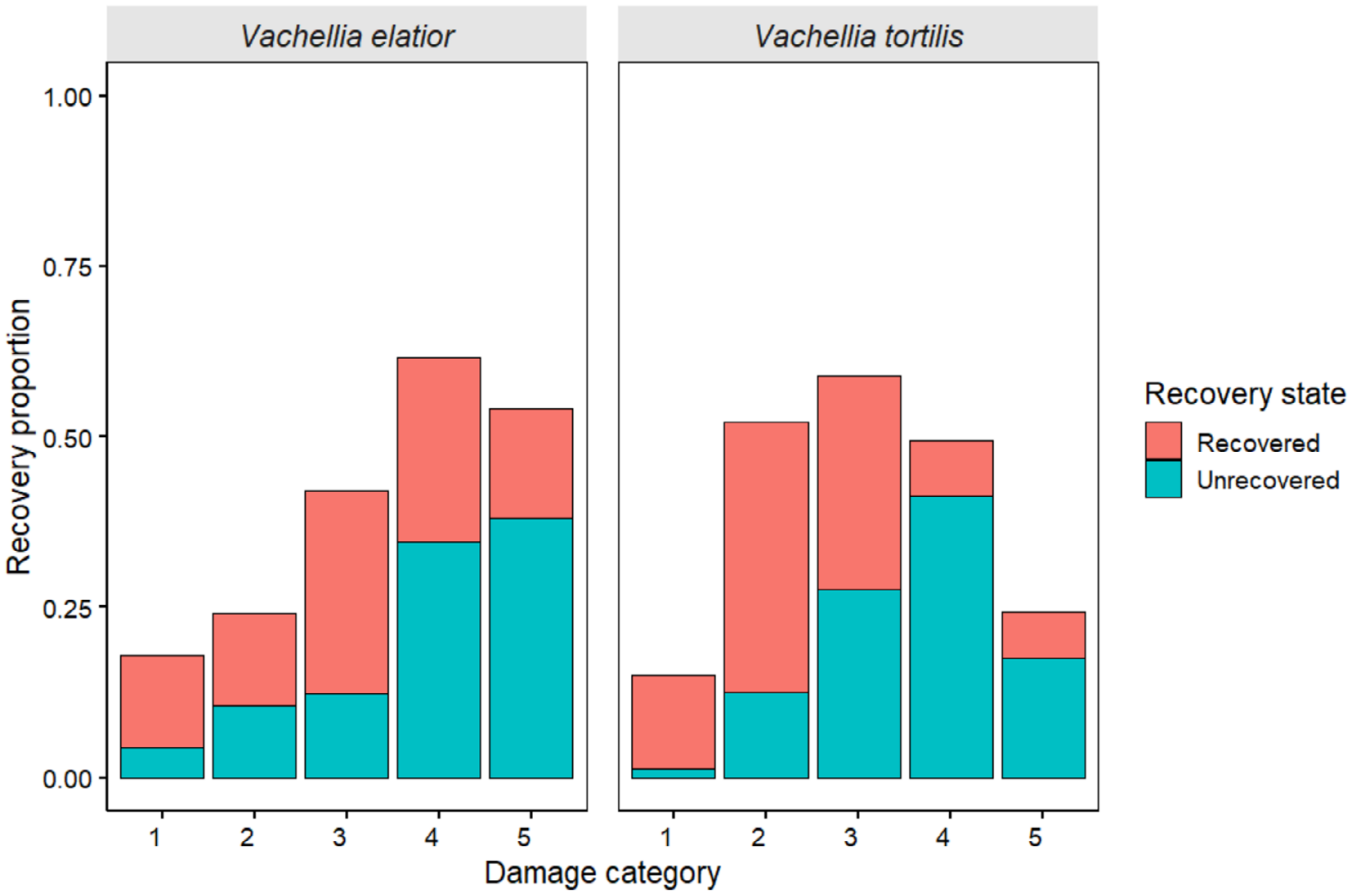
Proportion of recovered and unrecovered trees in *Vachellia elatior* (*n* = 422) and *Vachellia tortilis* (*n* = 153) across five debarking categories.

**FIGURE 11:**
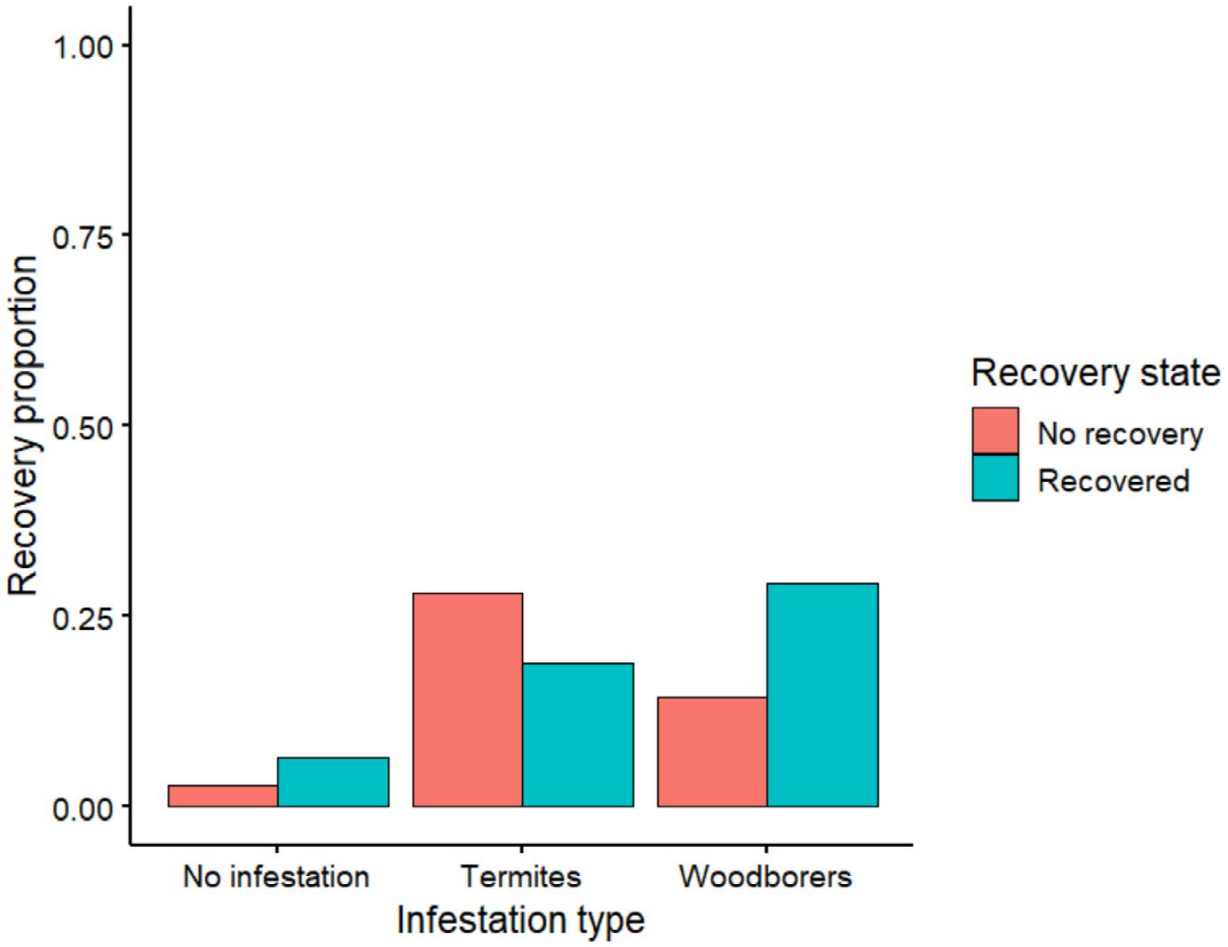
Recovery proportion of the two combined tree species under different infestation levels (no infestation, termite presence, or woodborer presence), showing the proportion of recovered and unrecovered trees.

Recovery responses following debarking showed species‐specific patterns among the riverine tree species (Figure S4), although statistical analysis was limited to *Vachellia elatior* and *Vachellia tortilis* due to insufficient sample sizes in the other species. These trees mainly responded to debarking through coppicing and bark regrowth (Figure S4). The two dominant and most preferred species, *Vachellia elatior* and *Vachellia tortilis* , were selected for recovery modeling. Based on AIC, two models were ranked highest (∆AIC < 2; Table 2). The best‐supported model included damage category, insect presence, and stem decay (AIC = 456.9, weight = 0.623; Table 2). The second‐ranked model included the same predictors plus circumference size (AIC = 458.9, ΔAIC = 2.02, weight = 0.227; Table 2). Together, these two models accounted for 85% of the cumulative model weight, indicating strong support relative to all competing models. All other candidate models had substantially lower support (∆AIC > 4). The recovery response of the two common tree species was significantly influenced by the extent of debarking (Table 1). Trees with low to moderate damage exhibited the highest recovery, whereas heavily damaged trees showed markedly lower recovery, with both species showing a decline in recovery and a corresponding increase in the proportion of unrecovered individuals as damage severity increased (Figure 10). Additionally, trees affected by termite infestation had a lower proportion of recovery than woodborer‐affected trees (Figure 11), indicating a notable impact of termite presence on post‐debarking regeneration. In contrast, the presence of woodborers had no statistically significant effect on tree recovery (Table 1). Additionally, trees exhibiting signs of stem decay demonstrated a significantly reduced likelihood of recovery, with stem decay negatively influencing the regeneration process (Table 1).

**TABLE 1:** GLMM outputs of dependent (recovery response) and independent variables (fixed effects) for the two most common woody species tested.

Variable	Fixed effects	Estimate	Std. error	*Z* value	*p*
	(Intercept)	0.2361	0.6167	0.383	0.70177
Recovery response	Damage category 2	1.6735	0.5622	2.977	0.00291
	Damage category 3	1.1230	0.5630	1.995	0.04606
	Damage category 4	1.6072	0.5744	2.798	0.00514
	Damage category 5	0.4134	0.5906	1.7000	0.48402
	Insect present (Termites)	−1.1990	0.3882	−3.089	0.00201
	Insect present (Woodborers)	0.3498	0.3443	1.016	0.30963
	Stem decay (Yes)	−0.8176	0.3330	−2.455	0.01407

**TABLE 2:** Top GLMM outputs from the 95% confidence set based on AICc weights, computed using the confset() function from the AICcmodavg package with the raw method. The top two models explain 85% of the model support for recovery response.

Fixed effects	*K*	AICc	Delta AICc	Cum.Wt
Damage category + Insect present + Stem decay	10	456.9	0.00	0.623
Damage category + Stem decay + Insect present + Circumference size	11	458.9	2.02	0.230
Damage category + Insect present	9	461.3	4.32	0.07
Damage category + Infestation + Insect present*Stem decay	13	463.2	6.21	0.03
Damage category*Stem decay + Insect present + Infestation	14	463.5	6.58	0.02

## 4 Discussion

In this study, we compared the effects of African elephants on a riverine forest in northern Kenya between two surveys that were conducted 16 years apart, in 2007 and 2023, respectively. Supporting our hypothesis that elephants had exerted negative impacts on trees over this period, we found that tree density in the riverine forest of the Samburu and Buffalo Springs National Reserves had significantly declined in 2023 compared to 2007. This decline in density was particularly marked for one tree species ( *V. elatior* ) favored by elephants as a food source—suggesting that elephant activity was the main cause of the decline. Moreover, the number of trees across debarking categories was significantly lower in 2023 than in 2007. The density of *Vachellia tortilis* , another species favored by elephants, did not vary significantly between the two survey periods. However, contrary to our expectations, we also found that the number of tree species in the riparian forest was higher in 2023 relative to 2007. These findings suggested that the impact of elephants on the riverine forest was more complex than expected.

The finding that the density of *V. tortilis* had not declined over time should be interpreted with caution. The observed pattern may reflect the sparser distribution of this species in the riverine forest, with some plots lacking the species entirely (Figure 8). Notably, unlike *Vachellia elatior*, which is restricted to the riverine forest, *V. tortilis* exhibits a broader distribution, extending into areas farther from the river, with only a few individuals occurring along the riverine forest in certain sections.

The observed changes in tree density and species composition appear to be influenced by recruitment limitations and sapling survival, which we assessed in Samburu and Buffalo Springs. These changes may be driven in part by elephant activity, as the riverine zone serves as a dry‐season refuge and elephants exhibit strong preferences for certain woody plants (Ihwagi 2007). Environmental pressures, including riverbank degradation, drought, and limited regeneration, likely exacerbate this effect. In particular, we observed reduced recruitment potential in *Vachellia elatior* compared to the 2007 survey, which may reflect both the decline in mature trees and increased disturbance of saplings resulting from the growing elephant population. Although elephants can promote sapling recruitment through seed dispersal (Campos‐Arceiz and Blake 2011), this potential benefit is often offset by intense browsing pressure from other herbivores, including giraffes, gerenuks, and impalas, which are common in the reserves. Impalas, in particular, exert significant browsing pressure on saplings, thereby inhibiting their survival and growth (O'Kane et al. 2012). More broadly, herbivory has been shown to suppress sapling regeneration and establishment, especially when combined with environmental stressors such as fire, creating a demographic bottleneck that prevents saplings from maturing into adult trees (Staver et al. 2009).

As a consequence of these recruitment limitations, we observed an increase in the abundance of *Prosopis chilensis* , *Salvadora persica*, *Cordia sinensis* , *Boscia coriacea*, and *Lawsonia inermis* within this disturbed riverine area. This shift suggests a successional response, with these species establishing in gaps created by the loss of *V. elatior* . The proliferation of the invasive *P. chilensis* , in particular, may be facilitated by herbivore‐mediated seed dispersal. Although this study did not specifically assess the role of elephants or other herbivores in dispersal dynamics, their potential influence on community composition and successional pathways warrants further investigation.

Concurrent with changes in species composition and tree density, patterns of elephant debarking also appear to have shifted over time. However, we found that the number of trees across debarking categories was significantly lower in 2023 than in 2007 for *Vachellia elatior*. This was in contrast with our hypothesis that debarking would have been proportionally higher compared to the baseline survey, due to progressively greater elephant impacts on woody vegetation (O'Connor and Page 2014; Chakuya 2022). However, the reduced numbers of debarked *V. elatior* in the latter study likely do not reflect diminished debarking pressure, but rather represent an ecological consequence of prolonged selective foraging (Ihwagi 2007). The substantial decline in *V. elatior* density—driven by sustained herbivory, mortality of mature individuals, and limited regeneration—has reduced the pool of trees available for debarking. Consequently, even if debarking pressure has remained constant, or possibly intensified, it now exerts its effects on a diminished population, resulting in a lower representation of debarked individuals. This pattern suggests a density‐dependent feedback, whereby continued exploitation of a preferred forage species leads to progressive demographic depletion, ultimately altering elephant foraging dynamics and reshaping debarking patterns at the riverine forest.

The observed predominance of debarked trees within medium circumference classes (47–210 cm) in Samburu and Buffalo Springs is consistent with patterns documented in the 2007 baseline survey for these reserves (Ihwagi et al. 2010). Although studies in other ecosystems report varying size‐selection patterns—such as a decline in debarking frequency from smaller to larger stems in Rabongo forest (Odoi et al. 2016) and preferential targeting of larger stems in Kruger National Park due to higher bark biomass and nutrient content (Moncrieff et al. 2008)—these differences likely reflect context‐specific foraging strategies. Across these systems, elephants appear to optimize bark foraging by balancing nutritional gain, accessibility, and handling effort. Within Samburu and Buffalo Springs National Reserves, medium‐sized trees likely represent an optimal foraging target, offering both sufficient nutritional reward and structural accessibility. In contrast, smaller stems provide limited bark and are more fragile, whereas larger stems possess tougher tissues that are more resistant to bark removal (Ihwagi 2007). Collectively, these observations indicate that tree circumference is a key determinant of susceptibility to elephant bark‐stripping, reflecting foraging efficiency and energetic optimization.

In Samburu, post‐disturbance recovery mechanisms were largely consistent across species, with the majority of individuals exhibiting coppicing and bark regrowth following debarking (Figure S4). Comparable regeneration patterns have been documented in other woody plant taxa, including *Acacia brevispica* and *Grewia tembensis* (Chira and Kinyamario 2009). Nevertheless, interspecific variation in regenerative capacity has been widely reported. For instance, *Ficus* spp. demonstrates a pronounced ability to regenerate following injury, attributed to their high sprouting potential and inherent physiological resilience, whereas *Prunus africana* exhibits limited recovery due to its poor resprouting capacity once the cambium is compromised (Tweheyo et al. 2013). Furthermore, bark traits such as thickness, moisture content, and anatomical structure have been shown to influence post‐debarking recovery potential (Wigley et al. 2019). In the present study, individuals of *V. elatior* and *V. tortilis* species that exhibited restricted recovery frequently displayed bark detachment along wound margins, indicating localized tissue desiccation and suppression of regenerative processes.

Although the most common trees in Samburu responded to debarking through coppicing and bark regrowth, the success of these regenerative pathways varied with the severity of debarking, biological infestation, and wound condition. The extent of ring‐barking (circumferential bark removal) has been shown to exert a pronounced negative effect on canopy health, with complete girdling largely inhibiting recovery (Delvaux, Sinsin, and Van Damme 2010; Delvaux, Sinsin, van Damme, and Beeckman 2010; Leaver and Cherry 2020). However, we also found evidence for biological infestation as a significant determinant of post‐debarking recovery. Termite activity was notably higher in non‐recovering individuals, suggesting a suppressive effect on regeneration, particularly where wounds retained moisture and bark detachment had occurred. Comparable observations have been made in South Africa, where termite infestation was associated with reduced recovery in low‐fire environments (Thompson et al. 2022; Wigley et al. 2019). In Samburu, termite activity peaked shortly after the rainy season, likely driven by elevated stem moisture. Previous studies have demonstrated that termite colonization is facilitated by bark loss, increased physiological stress, and the formation of moist, decomposing substrates around wound sites (Thompson et al. 2022). Furthermore, high moisture availability and warm temperatures enhance termite infestation and colonization, whereas prolonged drought and intense solar exposure tend to limit activity, except where deep moisture persists (Cornelius and Osbrink 2011).

Although termite activity negatively affected the recovery of debarked trees, woodborers were frequently observed on trees showing signs of recovery and did not appear to inhibit bark regeneration. This observation aligns with findings from fenced reserves in South Africa, where regrowth occurred despite the presence of woodborers (Thompson et al. 2022). However, in other ecosystems, such as in Benin, woodborers have been associated with the introduction of fungal pathogens that delay recovery (Delvaux et al. 2009). These contrasting outcomes may reflect species‐specific interactions, potentially mediated by chemical cues such as ethanol or terpenoids emitted by wounded trees (Lieutier et al. 2004; Ranger et al. 2010). Although such mechanisms were not assessed in the present study, they may contribute to the increased vulnerability of debarked trees to secondary colonization by pathogens or insects.

A key limitation of this study came from the impossibility of species‐level identification of insects associated with debarked trees, due to field conditions. We recommend that future research address this gap by identifying termite and woodborer species, as their ecological roles and impacts on tree recovery likely vary. Understanding their trophic preferences—such as targeting live versus dead tissue—could clarify their influence on post‐debarking outcomes.

In summary, we found that a riverine forest in semi‐arid northern Kenya had changed in structure and composition over a 16‐year period. Elephants appeared as the main drivers of such changes, since most of the changes were restricted to one preferred elephant forage species ( *V. elatior* )—with a reduction in density of both trees and saplings. Nonetheless, other factors in addition to elephants—most notably termite infestation and wound decay—also inhibited recovery, further reducing the trees' ability to recover and highlighting the complex interplay between physical damage and biological agents in shaping vegetation dynamics. In light of these results, we recommend the establishment of long‐term monitoring programs that assess survival and recovery across size or age classes, particularly in zones where elephant impacts are concentrated. Additionally, future studies should investigate how the extent of debarking by elephants affects tree vulnerability and healing, as it may be a key factor influencing both insect colonization and recovery potential. Collectively, these insights highlight the need to consider elephant‐induced damage not only as an immediate mechanical impact but as a catalyst for broader ecological change.

## Author Contributions

**Vincent Kipkazi:** conceptualization (equal), data curation (lead), formal analysis (lead), methodology (equal), writing – original draft (lead), writing – review and editing (equal). **Eunice Kairu:** conceptualization (equal), formal analysis (equal), methodology (equal), supervision (lead), writing – review and editing (equal). **Giacomo D'Ammando:** writing – review and editing (equal). **George Wittemyer:** writing – review and editing (equal). **Iain Douglas‐Hamilton:** conceptualization (equal), funding acquisition (equal). **Festus W. Ihwagi:** conceptualization (equal), data curation (equal), formal analysis (equal), funding acquisition (lead), methodology (equal), software (equal), supervision (equal), writing – review and editing (equal).

## Funding

This study was supported by Save The Elephants, Masters Research Fund Scholarship.

## Conflicts of Interest

The authors declare no conflicts of interest.

## Supporting information


**Data S1:** ece372692‐sup‐0001‐Supinfo01.docx.

**Data S2:** ece372692‐sup‐0002‐Supinfo02.docx.
**Table S1:** Table showing the utilization classes of trees as circumference percentage at the height of worst damage, and the weighting factor for each class.
**Table S2:** The GLMM structure of the candidate models used for the best model selection of the dependent variable (Trees recovery response).
**Table S3:** GLMM outputs of dependent (Recovery response‐second best model) and independent variables (fixed effects) for the two most common woody species tested.
**Figure S1:** Residual diagnostic plots generated using the DHARMa package in R, showing no significant deviations from model assumptions for the generalized linear mixed model of tree recovery after debarking.
**Figure S2:** Percentage of trees in different debarking categories and the corresponding percentages of observed plant responses.
**Figure S3:** Percentage of trees showing varying levels of insect infestation (termites and woodborers) or no infestation.
**Figure S4:** Recovery to debarking responses shown by different debarked tree species after utilization by African elephants in Samburu and Buffalo Springs National Reserves. Sample sizes were: *Cordia sinensis* (*n* = 8), *Gardenia volkensii* (*n* = 4), *Kigelia africana* (*n* = 4), *Lawsonia inamis* (*n* = 10), *Prosopis chillensis* (*n* = 27), *Senegal senegalia* (*n* = 4), *Vachellia elatior* (*n* = 422), and *Vachellia tortilis* (*n* = 153).

## Data Availability

All the required data have been uploaded and are publicly available on the Dryad website (https://doi.org/10.5061/dryad.3xsj3txvx).
