# Supplementary material for: Elephant Driven Changes in Riverine Tree Density Exacerbated by Biological Infestation in Samburu and Buffalo Springs National Reserves, Kenya
Source: Ecol Evol. 2025 Dec 12;15(12):e72692. doi: 10.1002/ece3.72692 (PMC12700725; doi:10.1002/ece3.72692)
Supplement: Supplementary file 1 — Data S1: ece372692‐sup‐0001‐Supinfo01.docx. [file ECE3-15-e72692-s002.docx]

**Data Availability Statement**

All the required data have been uploaded as supplementary materials, with an open-access link to the data repository provided. (<https://drive.google.com/drive/folders/17EAMHqOr0DQxj3JmBHxbZbGj46tTEdxW?usp=drive_link>).
