# Supplementary material for: Elephant Driven Changes in Riverine Tree Density Exacerbated by Biological Infestation in Samburu and Buffalo Springs National Reserves, Kenya
Source: Ecol Evol. 2025 Dec 12;15(12):e72692. doi: 10.1002/ece3.72692 (PMC12700725; doi:10.1002/ece3.72692)

**Supporting information**

**Table S1:** Table showing the utilization classes of trees as circumference percentage at the height of worst damage, and the weighting factor for each class.

| Category | Class (Debarked stem proportion) |
| --- | --- |
| 0 | 0% |
| 1 | 1-25% |
| 2 | 26-50% |
| 3 | 51-75% |
| 4 | 76-99% |
| 5 | 100% |

**Table S2:** The GLMM structure of the candidate models used for the best model selection of the dependent variable (Trees recovery response).

| **Fixed effects** | **K** | **AICc** | **Delta AICc** | **ModelLik** | **AICcWt** | **LL** | **Cum.Wt** |
| --- | --- | --- | --- | --- | --- | --- | --- |
| Damage category + Insect present + Stem decay | 10 | 456.9 | 0 | 1 | 0.623 | -218.17 | 0.623 |
| Damage category + Stem decay + Insect present + Circumference size | 11 | 458.9 | 2.02 | 0.364 | 0.227 | -218.1 | 0.850 |
| Damage category + Insect present | 9 | 461.3 | 4.32 | 0.115 | 0.072 | -221.4 | 0.922 |
| Damage category + Infestation + Insect present*Stem decay | 13 | 463.1 | 6.208 | 0.045 | 0.028 | -218.06 | 0.950 |
| Damage category*Stem decay + Insect present + Infestation | 14 | 463.5 | 6.581 | 0.037 | 0.023 | -217.2 | 0.973 |
| Damage category + Infestation + Insect present*Stem decay + Circumference size | 14 | 465.2 | 8.266 | 0.016 | 0.010 | -218.0 | 0.983 |
| Damage category*Stem decay + Insect present + Circumference size | 15 | 465.6 | 8.656 | 0.013 | 0.008 | -217.1 | 0.991 |
| Damage category*Infestation | 11 | 466.8 | 9.864 | 0.007 | 0.004 | -222.0 | 0.996 |
| Damage category + Infestation | 7 | 466.9 | 10.0 | 0.006 | 0.004 | -226.3 | 0.999 |
| Insect Infestation | 5 | 477.4 | 20.4 | 3.69E-05 | 1.79E-05 | -233.6 | 0.999 |
| Damage category | 6 | 477.8 | 20.9 | 2.93E-05 | 1.83E-05 | -232.8 | 0.999 |
| Damage category + Stem decay | 7 | 479.8 | 22.89 | 1.07E-05 | 6.66E-06 | -232.8 | 0.999 |
| Infestation | 3 | 482.1 | 25.1 | 3.51E-06 | 2.19E-06 | -238.0 | 0.999 |
| Damage category* Stem decay | 11 | 484.8 | 27.9 | 8.78E-07 | 5.47E-07 | -231.1 | 1 |
| Stem decay | 3 | 497.9 | 41.04 | 1.23E-09 | 7.65E-10 | -245.9 | 1 |
| Circumference size | 3 | 498.1 | 41.12 | 1.18E-09 | 7.33E-10 | -246.0 | 1 |

**Table S3:** GLMM outputs of dependent (Recovery response-second best model) and independent variables (fixed effects) for the two most common woody species tested.

| **Variable** | **Fixed effects** | **Estimate** | **Std. Error** | **Z value** | **p value** |
| --- | --- | --- | --- | --- | --- |
|  | (Intercept) | 0.2415 | 0.6172 | 0.391 | 0.6955 |
| Recovery response | Damage category 2 | 1.6657 | 0.5629 | 2.959 | 0.00309 |
|  | Damage category 3 | 1.1162 | 0.5634 | 1.981 | 0.0476 |
|  | Damage category 4 | 1.6043 | 0.5746 | 2.792 | 0.0052 |
|  | Damage category 5 | 0.4257 | 0.5918 | 0.719 | 0.4719 |
|  | Insect present (Termites) | -1.2097 | 0.3901 | -3.101 | 0.0019 |
|  | Insect present (Woodborers) | 0.3549 | 0.3447 | 1.030 | 0.3031 |
|  | Equivalent circumference | -0.0402 | 0.1229 | -0.327 | 0.7435 |
|  | Stem decay (Yes) | -0.8209 | 0.3333 | -2.463 | 0.0138 |

**Figure S1:** Residual diagnostic plots generated using the DHARMa package in R, showing no significant deviations from model assumptions for the generalized linear mixed model of tree recovery after debarking.


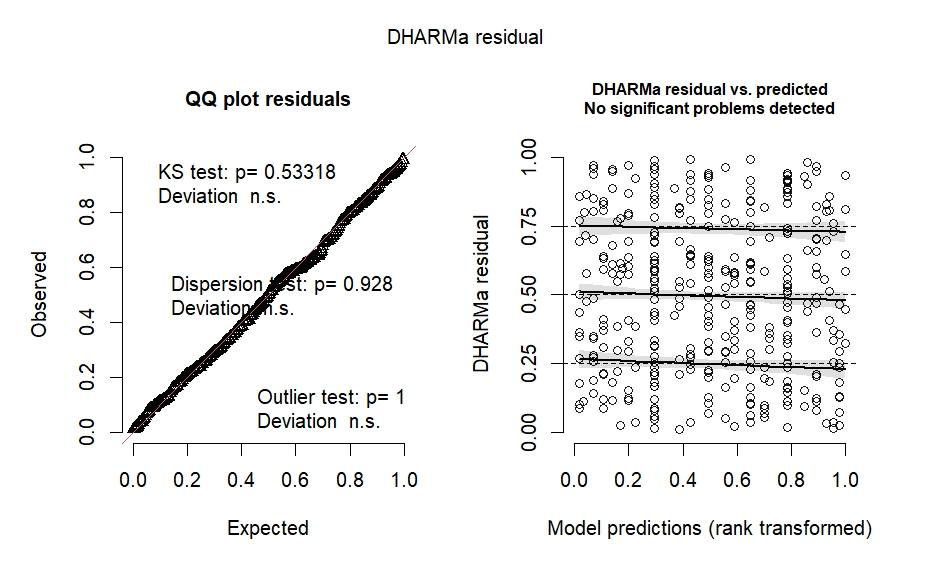


**Figure S2:** Percentage of trees in different debarking categories and the corresponding percentages of observed plant responses.


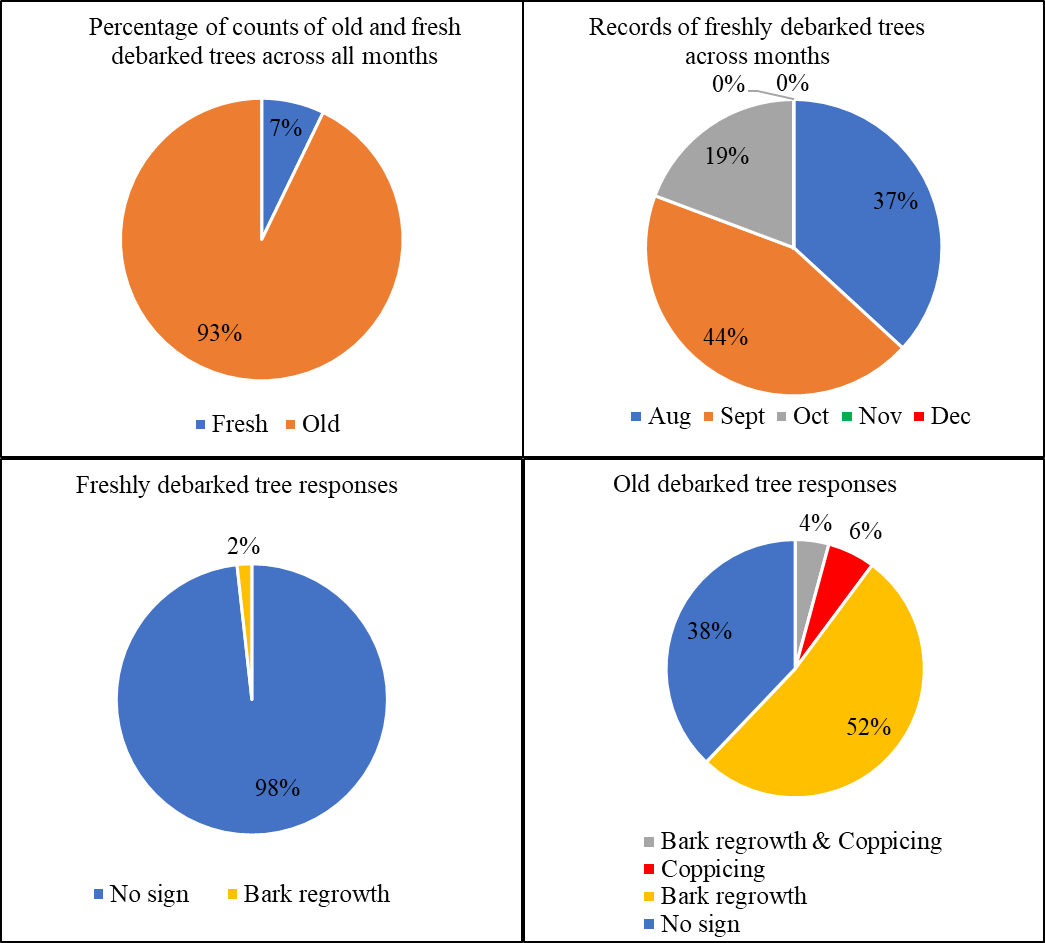


**Figure S3:** Percentage of trees showing varying levels of insect infestation (termites and woodborers) or no infestation. **
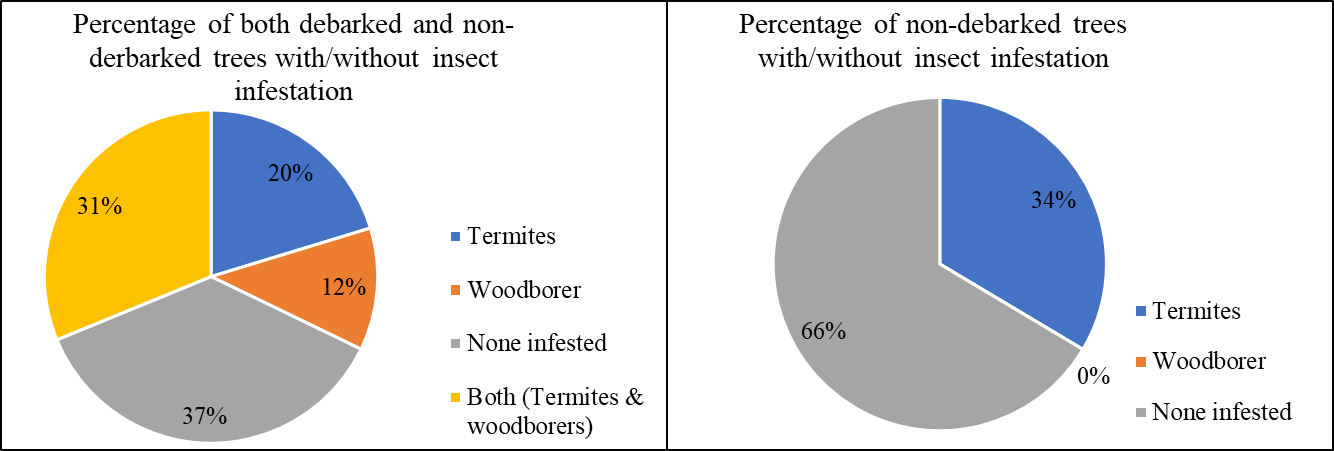
**

**Figure S4:** Recovery to debarking responses shown by different debarked tree species after utilization by African elephants in Samburu and Buffalo Springs National Reserves. Sample sizes were: *Cordia sinensis* (n = 8), *Gardenia volkensii* (n = 4), *Kigelia africana* (n = 4), *Lawsonia inamis* (n = 10), *Prosopis chillensis* (n = 27), *Senegal senegalia* (n = 4), *Vachellia elatior* (n = 422), and *Vachellia tortilis* (n = 153).


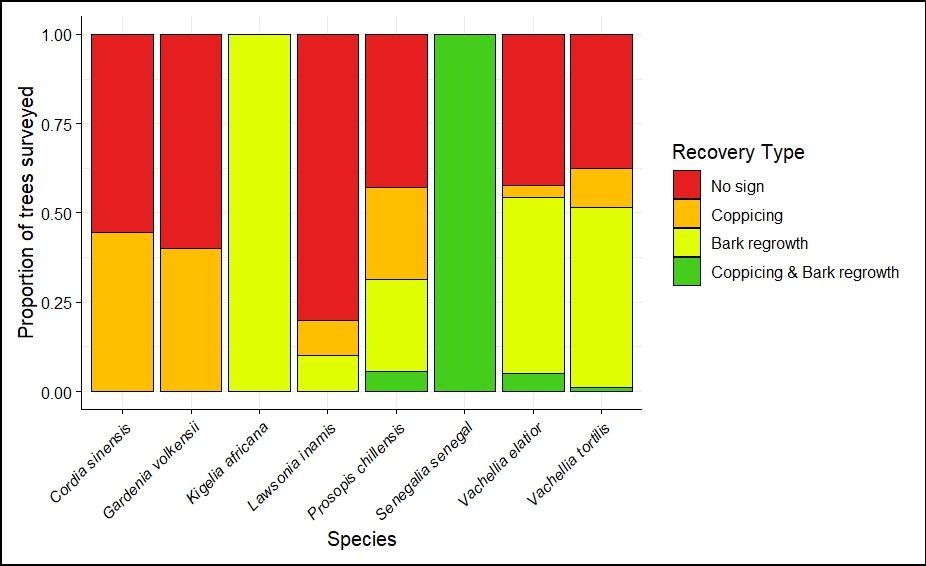

Supplement: Supplementary file 2 — Data S2: ece372692‐sup‐0002‐Supinfo02.docx. Table S1: Table showing the utilization classes of trees as circumference percentage at the height of worst damage, and the weighting factor for each class. Table S2: The GLMM structure of the candidate models used for the best model selection of the dependent variable (Trees recovery response). Table S3: GLMM outputs of dependent (Recovery response‐second best model) and independent variables (fixed effects) for the two most common woody species tested. Figure S1: Residual diagnostic plots generated using the DHARMa package in R, showing no significant deviations from model assumptions for the generalized linear mixed model of tree recovery after debarking. Figure S2: Percentage of trees in different debarking categories and the corresponding percentages of observed plant responses. Figure S3: Percentage of trees showing varying levels of insect infestation (termites and woodborers) or no infestation. Figure S4: Recovery to debarking responses shown by different debarked tree species after utilization by African elephants in Samburu and Buffalo Springs National Reserves. Sample sizes were: Cordia sinensis (n = 8), Gardenia volkensii (n = 4), Kigelia africana (n = 4), Lawsonia inamis (n = 10), Prosopis chillensis (n = 27), Senegal senegalia (n = 4), Vachellia elatior (n = 422), and Vachellia tortilis (n = 153). [file ECE3-15-e72692-s001.docx]
